# Echocardiographic Findings and Their Impact on Outcomes of Critically Ill Patients with AIDS in the Era of HAART

**DOI:** 10.1155/2012/575793

**Published:** 2012-04-09

**Authors:** Abubakr A. Bajwa, James D. Cury, Lisa Jones, Adil Shujaat, Faisal Usman

**Affiliations:** Shands Hospital, College of Medicine, University of Florida, 655 West 8th Street, Jacksonville, FL 32209, USA

## Abstract

*Objective*. To describe the echocardiographic findings in critically ill patients with AIDS and their impact on clinical outcome. *Design*. A retrospective chart review of consecutive AIDS patients over 18 years of age, who had a trans-thoracic echocardiogram performed during the course of intensive care unit stay over the course of 2 years at a tertiary care hospital. *Main outcome measures*. The prevalence of echocardiogram abnormalities in this population and its impact on ICU mortality, ICU length of stay, hospital mortality, hospital length of stay and 60 day survival. *Results*. Among 107 patients who met the inclusion criteria, an admission echocardiogram was performed in 62 (58%). The prevalence of cardiac abnormalities was 60%. The most common admission diagnosis was respiratory failure *n* = 27 (43%). The most common finding on echocardiogram was left ventricular (LV) dysfunction *n* = 31 (50%) followed by pulmonary hypertension *n* = 25 (40%). None of these findings had a significant impact on clinical outcomes. There was trend toward reduced 60 day survival among patients with depressed LV function. *Conclusions*. Although echocardiogram abnormalities were prevalent among this population none of these findings had a significant impact on ICU mortality or hospital mortality and ICU length of stay or hospital length of stay.

## 1. Introduction

The CDC estimates that 56,300 new HIV infections occurred in the United States in 2006 [1]. In the era of highly active antiretroviral therapy the mortality from HIV has been decreasing [2–6]. More patients are admitted to the intensive care unit for non-AIDS-associated illnesses than in the past [2, 5–8]. Advances in echocardiogram technology have provided the critical care physicians with a reliable and noninvasive method of determining cardiac function and chamber sizes and to detect the presence of valve diseases and pericardial disease. Due to its ready availability and noninvasive nature, echocardiography is commonly used in the critically ill population. A number of echocardiogram findings have been described in the noncritically ill HIV population with the most common ones being pericardial effusion, myocarditis, dilated cardiomyopathy, endocarditis, and pulmonary hypertension, while a recent prospective observational study showed that 18% of the HIV patients had systolic, 26% had diastolic dysfunction, and up to 57% had evidence of pulmonary hypertension based on TR jet velocity [9, 10]. Pericardial effusion has also been shown to predict increased mortality among HIV infected patients [11, 12]. Blanc et al. performed a prospective echocardiographic study in HIV-patients admitted to the intensive care unit (ICU) preceding the highly active antiretroviral therapy (HAART) era and found a similar prevalence of cardiac abnormalities as previously published studies [13]. Hakim et al. presented an abstract of echocardiographic findings in hospitalized HIV patients which showed that 50% of the patients have echocardiographic abnormalities [14]. The prevalence of pulmonary hypertension in HIV-infected patients has changed little from the 1990s (0.5%), when HAART therapy was not readily available to the 2000s (0.46%), when HAART therapy is easily available [15, 16]. The prevalence of pulmonary hypertension in critically ill patients with AIDS is not known.

Acquired immunodeficiency syndrome (AIDS) represents the most advanced part of the spectrum of HIV infected population. The CDC estimates that in 2006 there were 436,693 persons living with AIDS in the United States. More AIDS patients get admitted to the intensive care unit due to the changing spectrum of management in this era due to availability of HAART therapy. Afessa et al. showed that in their center higher APACHE II scores and transfer from another hospital were associated with poor outcome [4] however like most other studies, this study included patients with HIV infection with or without AIDS. To date no studies have described echocardiographic findings in critically ill patients with AIDS in the HAART era and their impact on clinical outcomes.

## 2. Methods

After approval by the institutional review board, we performed a retrospective analysis of consecutive AIDS patients admitted to the medical intensive care unit of an urban tertiary care center from 2005–2008. Patients who did not have an echocardiogram done during ICU admission were excluded from the analysis. We collected demographics data, admission diagnoses and calculated the Acute Physiology and Chronic Health Evaluation (APACHE 2) Score. We also documented ICU mortality, hospital mortality, and 60-day survival. The use of HAART therapy, compliance with HAART therapy, CD4 count, and HIV viral loads were also recorded. The echocardiographic findings were documented after a review of the echocardiogram reports. We also documented the presence and severity of pericardial effusion, right atrial dilation, left atrial dilation and size in cm, evidence of pulmonary hypertension as defined by TR jet velocity greater than 2.5 meters per second (m/sec), estimated pulmonary artery systolic (PAS) pressure, TR jet velocity in m/s, right ventricular (RV) dilation, RV dysfunction, left ventricular (LV) dilation, LV end systolic (ES) and end diastolic (ED) size in cm, LV ejection fraction (EF), and LV fractional shortening in percentage. LV ejection fraction measurements from echocardiogram results were categorized as normal (≥55%), mildly depressed (45%–54%), moderately depressed (35%–44%), and severely depressed (<35%).We performed a descriptive and comparative analysis of the data. Mean and range or standard deviation was used to describe normally distributed continuous data. Median and interquartile range (IQR 25–75) was used to describe nonnormally distributed data, and percentages were used to describe categorical data. A logistic regression analysis was performed to evaluate the impact of multiple echocardiogram parameters on ventilator days, ICU mortality, hospital mortality, and length of stays both in the ICU and the hospital. A *P* value less than  .05 was considered statistically significant. A logistic regression analysis was performed to analyze the relationship between variables. Statistical analysis was performed using JMP (Statistical Discovery by SAS, Cary, NC, USA).

## 3. Results

**Figure 1:**
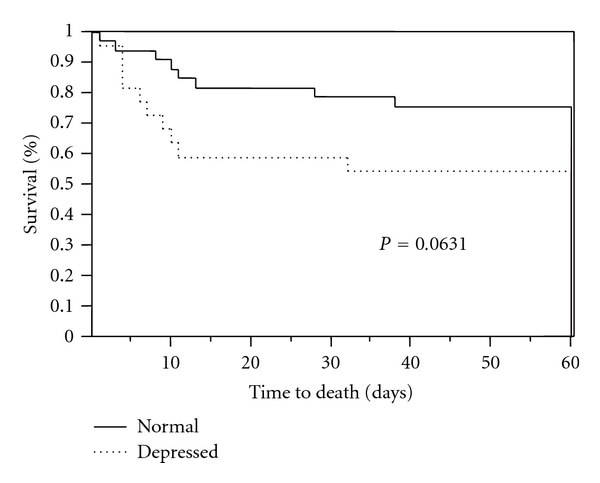
Kaplan-Meier survival curve for patients with echocardiographic findings of depressed LV function and normal LV function.

From 2005 to 2008, there were 107 AIDS patients admitted to the medical ICU at our institute. 62 (58%) of these patients had at least one echocardiogram done upon their admission to the ICU and were included in the analysis. Demographics are described in Table 1. The majority of the patients were male (70%) and black (90%). Only a small number of these patients was on HAART therapy *n* = 19 (31%), and only 10 of 19 (53%) were compliant with therapy based on documentation upon admission to the ICU. The most common diagnosis was respiratory failure *n* = 27 (43%) (Table 2). Mechanical ventilation was instituted in 43 of 62 (68%) patients with median ventilator days of 3 (IQR 2–7). The report of the first echocardiogram done during the ICU stay was reviewed, and the predefined data were collected. The echocardiogram was considered abnormal in 38 patients (60%), with the most common abnormality being left ventricular dysfunction *n* = 31 (50%) followed by evidence of pulmonary hypertension *n* = 25 (40%) and pericardial effusion *n* = 14 (22%). Although LV dysfunction was seen in 31 patients, only 22 of these patients had depressed function, one had diastolic dysfunction and the rest had hyperkinetic function (Table 3). In patients in whom there was evidence of pulmonary hypertension, only one patient had a right-heart catheterization done for management; however, no specific pulmonary hypertension treatment was instituted. Among patients who had pericardial effusions, none had evidence of tamponade or required pericardial drainage. The majority of the abnormalities noticed on echocardiograms were in patients with an admission diagnosis of respiratory failure (39%) followed by sepsis (18%). Among the 25 patients with pulmonary hypertension per echocardiogram, 9 (36%) had respiratory failure, 3 (12%) had pneumonia, mechanical ventilation was instituted in 16 (64%), and concomitant LV dysfunction was noticed in 14 (56%) of these patients. Although a number of echocardiogram abnormalities were prevalent in this population, on logistic regression analysis none of these findings had a significant impact on mortality, ventilator days and length of stay in the ICU or hospital. Although not significant, there was a trend towards reduced 60-day survival in patients with depressed LV function compared to those with normal LV function (Figure 1). No correlation was found between the presence of echocardiogram abnormalities and CD4 counts or viral loads. 

## 4. Discussion

This is the first study to look at the echocardiogram findings and its impact on clinical outcomes in critically ill patients with AIDS in the era of HAART. Advances in critical care medicine over the last two decades have resulted in improved outcomes of patients with HIV infection and AIDS. Echocardiography these days is done on almost every patient admitted to the intensive care units. The information obtained from the echocardiogram does guide treatment in majority of these patients and HIV patients are not different [13]. The first cardiac manifestation of AIDS was described in 1983 by Autran et al., when they published a case of cardiac Kaposi' sarcoma in a Haitian woman [17]. Since then, a number of studies have estimated the prevalence of cardiac involvement in AIDS patients to range from 28% to 73% [9, 11–13]. Echocardiographic abnormalities among hospitalized HIV population are reported to be around 50% in two different studies [13, 14]. The prevalence of abnormal echocardiogram in our AIDS population was slightly higher than previously reported for HIV population admitted to the ICU by Blanc et al. [13]. In the study by Blanc et al., pericardial effusion was the most common (29%) abnormality followed by LV dysfunction (22%). In the same study vegetations were noted in 3%, and sepsis was the leading cause of admission to the ICU (90%). In our study LV dysfunction (50%) and pulmonary hypertension (40%) were the most common abnormalities. No vegetations were noted. The higher prevalence of LV dysfunction in this group may reflect the availability of HAART, longer life span of AIDS patients, and acquiring the infection at a later age. However in our cohort, only 30% of our patient population was actually on drugs, and even a smaller number were actually compliant with therapy, making it possible that the lack of adequate control of HIV infection may be the cause of the critical illness and higher prevalence of echocadiographic abnormalities. Another factor to be considered is the presence of sepsis at the time of admission in our population. Myocardial dysfunction has been well documented in sepsis the spectrum of dysfunction ranges from hyperkinetic function to depressed EF [18]. It is difficult to ascertain whether these abnormalities were related to the underlying critical illness or to HIV infection itself. We were able to compare echocardiograms done during critical illness to a prior echocardiogram in 23 (36.5%) of the cases and almost all of them had similar abnormalities documented. Due to the design of the study, we were unable to evaluate whether or not these abnormal findings on echocardiogram resulted in any change in management strategy. Another noteworthy finding was the high prevalence of moderate pulmonary hypertension by echocardiogram (40%) in this population. The prevalence of pulmonary hypertension in noncritically ill HIV population ranges from 0.03% to 7% in different eras of HIV treatment and from various regions of the world [19–22]. Although right heart catheterization remains to be gold standard for diagnosis of pulmonary arterial hypertension, an echocardiogram has been routinely used in studies to screen for pulmonary hypertension in otherwise healthy HIV patients. In this critically ill population the presence of pneumonia, respiratory failure, mechanical ventilation along with high number of concomitant LV dysfunction rather than true pulmonary arterial hypertension, likely explain the high prevalence of pulmonary hypertension noticed on echocardiogram.

There are several limitations to this study. The retrospective nature limits the ability to derive any conclusion in regards to whether the findings noticed on echocardiogram lead to any management change during the course of ICU stay and hence limiting our ability to determine the impact of these findings on clinical outcome. There was also no way to determine whether standardized echocardiography techniques were uniformly employed to obtain images.

Based on our review, there was no prognostic significance of any single echocardiogram abnormality in this AIDS population. Whether an echocardiogram performed in the ICU in this critically ill AIDS population leads to findings that can significantly impact management decision remains to be seen.

## Figures and Tables

**Table 1:** Demographics, length of stay, and mortality.

Age, yrs. mean ± SD	47 ± 9
Sex	
Male, number (%)	44 (70)
Female, number (%)	18 (29)
Race	
Black, number (%)	56 (90)
Caucasian, number (%)	6 (10)
HAART therapy	
Yes	19 (31)
No	43 (70)
CD4 count, mean ± SD	95 ± 141
HIV viral load, copies/mL. mean ± SD	326270 ± 561689
APACHE II score, mean ± SD	23.4 ± 9.3
APACHE II predicted mortality, mean ± SD	47 ± 26
ICU LOS, median (IQR 25–75)	3 (2–7)
Hospital LOS, median (IQR 25–75)	10 (5–19)
Ventilator days, median (IQR 25–75)	3 (2–7)
ICU mortality, number (%)	21 (33)
Hospital mortality, number (%)	21 (33)

LOS: length of stay; HAART: highly active antiretroviral therapy.

**Table 2:** Admission diagnosis.

Diagnosis	Number (%)
Respiratory failure	27 (43)
Altered mental status	9 (14)
Sepsis	9 (14)
Pneumonia	4 (7)
Seizure	4 (7)
GI bleed	2 (3)
Ascites	2 (3)
Spinal cord compression	1 (1.6)
Diarrhea	1 (1.6)
Pneumothorax	1 (1.6)
Syncope	1 (1.6)
Paraplegia	1 (1.6)

**Table 3:** Echocardiogram findings.

Pericardial effusion, number (%)^†^	14 (22)
Moderate size	1 (7)
Small size	13 (93)
Pulmonary HTN, number (%)^†^	25 (40)
Estimated PA systolic pressure mm Hg, mean ± SD	45 ± 12
TR jet velocity m/sec, mean ± SD	2.9 ± 0.3
RA dilatation, number (%)^†^	16 (26)
Mild	14 (87)
Marked	2 (13)
Right ventricular enlargement, number (%)^†^	11 (18)
Mild	9 (82)
Moderate	2 (18)
Right ventricular dysfunction, number (%)^†^	2 (4)
Mild	1 (50)
Severe	1 (50)
LA size in cm, mean ± SD	3.7 ± 0.7
LA dilation, number (%)^†^	19 (31)
Mild	11 (58)
Moderate	6 (32)
Severe	2 (10)
LV size in cm, mean ± SD	
End systolic	3.4 ± 1.1
End diastolic	4.8 ± 0.8
LV ejection fraction (LVEF) %, mean ± SD	51 ± 19
LV fractional shortening %, mean ± SD	33 ± 11
LV dysfunction, number (%)^†^	31 (50)
Mildly depressed LVEF	6 (19)
Moderately depressed LVEF	8 (26)
Severely depressed LVEF	8 (26)
Hyperkinetic	8 (25)
Diastolic dysfunction	1 (3)

PA: pulmonary artery, TR: tricuspid regurgitation, LA: left atrium, and LV: left ventricle.

^†^Some patients had more than one echocardiographic finding.
